# Efficient population record linkage with temporal and spatial constraints.

**DOI:** 10.23889/ijpds.v7i3.1857

**Published:** 2022-08-25

**Authors:** Alexandra Procter, Catherine Chittleborough, Rhiannon Pilkington, John Lynch

**Affiliations:** 1 The University of Adelaide

## Objectives

Socioeconomic disadvantage and poverty are considered important drivers of child maltreatment, particularly child neglect. We examine the prevalence, cumulative incidence, and notification rate of child protection (CP) contact among children who experienced intergenerational welfare contact (IWC) compared to families with other types of welfare contact (WC).

## Approach

A whole-population linked administrative data study of children in South Australia born 1991-1995 (n=94,358), followed from birth up to age 20 years and their parent/s (n=143,814). The study used de-identified data from the Better Evidence Better Outcomes Linked Data (BEBOLD) platform including data from Australian Government Centrelink (welfare payments), birth registration, perinatal birth records, and child protection records. Children were classified as: No WC, parent only WC, child only WC, or IWC. Child protection contact prevalence, rates, and cumulative incidence were estimated by age, level of contact (reported notifications, screened-in reports, investigations, substantiations and out-of-home care), and WC group.

## Results

Using Australian Government Centrelink data, WC was defined as parent/s receiving a means-tested welfare payment (low-income, unemployment, disability or caring) when children were aged 11-15, or children receiving payment at ages 16-20. IWC was WC occurring in both parent and child generations. Children who experienced IWC accounted for 84.7% of all CP notifications at ages 0-17 years and had the highest prevalence of CP contact (37.3%, n=12,286), compared to children experiencing parent only WC (16.1%), child only WC (11.6%), and no WC (4.7%). This trend was consistent across all levels of CP contact. Children who experienced IWC had higher notification rates at every age compared to all other WC types.

## Conclusion and Relevance

Children experiencing IWC represent a third of the population aged 11-20, yet account for 84.7% of all child protection notifications. Our study shows that children who experience IWC have higher personal (cumulative incidence and prevalence) and societal (notification rates) child protection costs than their counterfactual (parent only WC).

